# FECAL CALPROTECTIN AND INTESTINAL METABOLITES: WHAT IS THEIR IMPORTANCE IN THE ACTIVITY AND DIFFERENTIATION OF PATIENTS WITH INFLAMMATORY BOWEL DISEASES?

**DOI:** 10.1590/0102-6720202500001e1870

**Published:** 2025-02-28

**Authors:** Lucas Correia LINS, Júnia Elisa Carvalho DE-MEIRA, Camila Wanderley PEREIRA, Alessandre Carmo CRISPIM, Marina Demas Rezende GISCHEWSKI, Manoel Álvaro de Freitas LINS-NETO, Fabiana Andréa MOURA

**Affiliations:** 1 Universidade Federal de Alagoas, Postgraduate Program in Medical Sciences - Maceió (AL), Brazil; 2 Universidade Federal de Alagoas, University Hospital Professor Alberto Antunes - Maceió (AL), Brazil; 3 Universidade Federal de Alagoas, Faculty of Medicine - Maceió (AL), Brazil; 4 Universidade Federal de Alagoas, Postgraduate Program in Chemistry and Biotechnology - Maceió (AL), Brazil

**Keywords:** Crohn Disease, Colitis, Ulcerative, Gastrointestinal Microbiome., Doença de Crohn, Colite ulcerativa, Microbioma Gastrointestinal.

## Abstract

**BACKGROUND:**

Inflammatory bowel disease (IBD), comprising Crohn’s disease (CD) and ulcerative colitis (UC), lacks a known etiology. Although clinical symptoms, imaging, and colonoscopy are common diagnostic tools, fecal calprotectin (FC) serves as a widely used biomarker to track disease activity. Metabolomics, within the omics sciences, holds promise for identifying disease progression biomarkers. This approach involves studying metabolites in biological media to uncover pathological factors.

**AIMS:**

The purpose of this study was to explore fecal metabolomics in IBD patients, evaluate its potential in differentiating subtypes, and assess disease activity using FC.

**METHODS:**

Cross-sectional study including IBD patients, clinical data, and FC measurements (=200 μg/g as an indicator of active disease).

**RESULTS:**

Fecal metabolomics utilized chromatography mass spectrometry/solid phase microextraction with MetaboAnalyst 5.0 software for analysis. Of 52 patients (29 UC, 23 CD), 36 (69.2%) exhibited inflammatory activity. We identified 56 fecal metabolites, with hexadecanoic acid, squalene, and octadecanoic acid notably distinguishing CD from UC. For UC, octadecanoic and hexadecanoic acids correlated with disease activity, whereas octadecanoic acid was most relevant in CD.

**CONCLUSIONS:**

These findings highlight the potential of metabolomics as a noninvasive complement for evaluating IBD, aiding diagnosis, and assessing disease activity.

## RESUMO

**RACIONAL:**

A doença inflamatória intestinal (DII), que engloba a doença de Crohn (DC) e a colite ulcerativa (CU), carece de etiologia conhecida. Embora sintomas clínicos, imagens e colonoscopia sejam ferramentas diagnósticas comuns, a calprotectina fecal (CalF) atua como biomarcador amplamente utilizado para monitorar a atividade da doença. A metabolômica, no âmbito das ciências “omics,” apresenta promissoras perspectivas para identificação de biomarcadores de progressão da doença. Este método envolve o estudo de metabólitos em meios biológicos para revelar fatores patológicos.

**OBJETIVOS:**

Explorar a metabolômica fecal em pacientes com DII, avaliando seu potencial na diferenciação de subtipos e na avaliação da atividade da doença usando CalF.

**MÉTODOS:**

Estudo transversal incluindo pacientes com DII, dados clínicos e medições de CalF (=200μg/g como indicador de doença ativa).

**RESULTADOS:**

A metabolômica fecal utilizou cromatografia de massa/extração em fase sólida com o software MetaboAnalyst 5.0 para análise. Dos 52 pacientes (29 CU, 23 DC), 36 (69,2%) apresentaram atividade inflamatória. Foram identificados 56 metabólitos fecais, sendo os ácidos hexadecanoico, esqualeno e octadecanoico notáveis na distinção entre CD e CU. Para CU, os ácidos octadecanoico e hexadecanoico correlacionaram-se com a atividade da doença, enquanto o ácido octadecanoico foi mais relevante em CD.

**CONCLUSÕES:**

Os achados destacam o potencial da metabolômica como um complemento não invasivo para avaliar a doença inflamatória intestinal, auxiliando no diagnóstico e na avaliação da atividade da doença.

## INTRODUCTION

Crohn’s disease (CD) and ulcerative colitis (UC), entitled inflammatory bowel diseases (IBD), are chronic diseases that cause inflammation in the gastrointestinal tract and extraintestinal complications, which share similar symptoms like abdominal pain, diarrhea, and weight loss, but differ in their extent, location, complications, and prevalence^26,28^.

Because of its impact on patients’ quality of life and work activities, IBD represents a significant public health problem. Treatment aims to achieve remission through a multifaceted approach that includes both nonpharmacological interventions like psychological and nutritional support, and pharmacological interventions like immunosuppressants and anti-inflammatories, as well as traditional surgical interventions^32,36^. The costs for the health system are considered high and are due to multiple factors such as diagnostic, medications, hospitalization, surgeries, and absenteeism from work^8,16^.

The etiology of IBD is unknown, but the interaction between genetics, immunological, and environmental factors, such as microbiota, contribute to its development^16^. In this context, intestinal dysbiosis - the imbalance of the intestinal microbiota - is closely related to the pathogenesis and clinical complications of IBD; however, it is still not possible to distinguish whether this dysbiosis is a cause or a consequence of the disease^2,38^. Thus, microbiota and dysbiosis are currently the subject of important investigations in the field of diagnosis and therapy in IBD.

The diagnosis of IBD typically involves invasive and expensive measures such as colonoscopy and histopathological evaluation, in addition to clinical assessment, which can be uncomfortable for patients. In recent years, studies have aimed to identify laboratory markers with high sensitivity and specificity for noninvasive diagnosis and monitoring of these diseases^7^.

Fecal calprotectin (FC), a calciumand zinc-binding protein found mainly within neutrophils, has been presented as an effective, low-cost, and highly reproducible marker for both diagnosis and follow-up of IBD^7^. FC correlates well with the top endoscopic assessment scores used in clinical practice [Simple Endoscopic Score for Crohn’s Disease (SES-CD), Ulcerative Colitis Score (UCIS), and Mayo Score)^34^. However, its low sensitivity (83%) and specificity (60%) make it a nonspecific inflammatory biomarker. Therefore, definitive diagnosis still requires biopsies collected through colonoscopy, which carries a risk of pain, bleeding, and perforation^7^.

Metabolomics, an omic technique, has been recently studied in several diseases, including IBD. It has been considered a high sensitivity and specificity method for assessing endoscopic activity using an FC cutoff of 200 μg/g of stool. Additionally, several metabolites identified by metabolomic techniques, such as tricarboxylic acid cycle (TCA) cycle intermediates, amino acids, fatty acids, glycerophospholipids, glycerolipids, prenol lipids, sterol lipids, choline, and sphingolipids in serum, urine, feces, or intestinal biopsies, are typically involved in molecular pathways implicated in the inflammatory response. This reinforces the diagnostic potential of metabolomic analysis^3^.

Despite increasing research on omic sciences in IBD, several questions remain unanswered, such as their use in the differential diagnosis of CD and UC, as well as their use in assessing the therapeutic potential of pharmacological and nonpharmacological treatments. Therefore, this study aims to identify metabolites present in the feces of individuals with IBD and correlate them with disease activity, as measured by levels of FC.

## METHODS

### Study design, participant selection, inclusion criteria, and ethical oversight

This study employs a cross-sectional design, with patient recruitment carried out via convenience sampling. The participant pool includes individuals of all genders and age ranges, diagnosed with IBD, who are under the care of the coloproctology outpatient clinic at University Hospital Professor Alberto Antunes, Universidade Federal de Alagoas, Brazil. Patients who accepted the invitation to participate in the study were provided with clear instructions regarding the proper collection of fecal samples. These samples were subsequently submitted during their subsequent clinic visit for the evaluation of both FC levels and fecal metabolites.

Conducting the study in strict accordance with the ethical principles set forth in the Declaration of Helsinki, it received formal endorsement from the Ethics and Research Committee of the Federal University of Alagoas (Approval Number: 2725386). This rigorous ethical oversight underscores the commitment to safeguarding the welfare and rights of all involved participants.

### Fecal calprotectin analysis

FC was analyzed using the BÜHLMANN Quantum Blue^®^ device. Stool samples were placed in extraction tubes and diluted with a 1:16 ratio of BÜHLMANN fCAL^®^ kit solution to extraction buffer. The resulting mixture was vortexed for 1 min and then centrifuged at 3000×g for 5 min. The final solution was subsequently analyzed using the instrument for a duration of 15 min. FC levels were classified as altered (high) when they reached=200 μg/g of feces^5^.

### Analysis of volatile organic compounds

Volatile organic compounds (VOCs) present in donors’ fecal samples were analyzed using gas chromatography mass spectrometry/solid phase microextraction (GC-MS/SPME) technique, as previously described^1^.

### Statistical analysis

The statistical analysis was performed using the SPSS^®^ version 26 software. Continuous variables were expressed as mean±standard deviation (SD) and categoric variables as frequency [n (%)]. Frequency comparison using the chi-square test/Fisher tests, according to CD/UC, and t-test was used to compare means. Significance was considered when the p<0.05.

The online platform MetaboAnalyst 5.0 (available free of charge at https://www.metaboanalyst.ca) was utilized to conduct all statistical analyses requiring computational support. This software, known for its widespread use and recommendation in this field, contributes to numerical precision and scientific reproducibility. Chromatogram data obtained through GC were transformed into a data matrix using Chromeleon 7.2 software (equipment program). Regions containing inferential signals were excluded from the analysis.

The concentrations of identified metabolites were normalized by the median, followed by logarithmic transformation (base 10) and scaling using the Pareto method. Supervised partial least squares discriminant analysis (PLS-DA) was conducted to discern metabolite variations among stool sample groups. PLS-DA, a supervised technique utilizing multivariate regression information, predicts class associations. Variations in sample metabolites were calculated using the variable importance on projection (VIP) measure. This score represents a weighted sum of squares of PLS loadings, accounting for the explained Y-variance of each component. In this study, VIP measurements above 2.0 were considered significant.

## RESULTS

Fifty-two patients with IBD, 29 with UC and 23 with DC, were studied. Of these patients, 38 were women and 14 were men, with a mean age of 41 years (UC=43.0±15.7, and CD=39.7±16.2; p=0.463, p>0.05), the youngest being 12 years old and the oldest being 69 years old. Thirty-six patients (69.2%) had high FC and had no statistical difference between the prevalence of inflammation detected by FC levels and the type of IBD (p>0.05). There was no significant difference in the overall demographic and clinical characteristics of the study participants concerning the type of IBD, as indicated in Table 1.

**Table 1:** Demographic and clinical characteristics according to inflammatory bowel disease source.

		Total n (%)	Ulcerative colitis n (%)	Chron disease n (%)	p-value
Sex	Female	38 (73.1)	22 (75.9)	16 (69.6)	0.611
	Male	14 (26.9)	7 (24.1)	7 (30.4)	
Race	Not white	47 (90.4)	27 (93.1)	20 (87.0)	0.644
	White	5 (9.6)	2 (6.9)	3 (13.0)	
Smoking	Yes	5 (9.6)	4 (13.8)	1 (4.3)	0.368
	No	47 (90.4)	25 (86.2)	22 (95.7)	
Alcohol consumption	Yes	5 (9.6)	1 (3.4)	4 (17.4)	0.157
	No	47 (90.4)	28 (96.6)	19 (82.6)	
Comorbidities	Yes	24 (46.2)	12 (41.4)	12 (52.2)	0.438
	No	28 (53.8)	17 (58.6)	11 (47.8)	
Surgery	Yes	25 (48.1)	15 (51.7)	10 (43.5)	0.544
	No	27 (51.9)	14 (48.3)	13 (56.5)	
Extraintestinal manifestations	Yes	35 (67.3)	18 (62.1)	17 (73.9)	0.366
	No	17 (32.7)	11 (37.9)	6 (26.1)	

Values are presented as numbers (%).

Seventy-four metabolites were found through the mass spectrometry library (Chromeleon 7.2). Metabolites were compared with data in the Human Metabolome Database (available at https://hmdb.ca), 18 of which were unidentified (Unknown). The 56 identified metabolites were: p-cresol, 2-undecanone, undecanal, indoleacetic acid, ethyl decanoate, ethyl-heptane, squalene, 1-decene, naphthalene, pentadecane, trimethyl-dodecathyene, tetradecanal, dodecanoic acid, dodecanoic-ethyl acid -ester, methyl amine, hexadecane, cyclooctasiloxane, dodecanol, trimethyl amine, pentadeicin, pentadecanone, nonadecene, tridecan-1-ol, tricosene, tetradecanoic acid, undecanone, pentadecanoic acid, hexadecanol, heptadecanone, squalene, pentadecanal, cyclodecasiloxone, docosene, hexadecanoic acid (palmitic acid), hexanoic acid, 9-octadecenal, cis-11-hexadecenal, octadecenal, 9-Octadecen-1-ol, linoleoyl chloride, octadecanol, oleic acid, cis-9-hexadecenal, octadecanoic acid (stearic acid), 11-hexadecen-1-ol, cyclononasiloxane, 1-hexadecanol, cyclopentadecane, heptacosane, cyclodecasiloxane, octadecane, docosane, 11-butyl-, quantacure ITX, heptacosane, and octacosane.

Figure 1 shows the result of the partial PLS-DA of samples between DC and UC. The points in Figure 1 represent the metabolic profile of each patient. Thus, patterns of separation can be seen in the data sets. There are patients with distinct profiles between UC and CD, and some in intersection areas where there are metabolites with variations in common between the two groups. The method has a good accuracy (0.60) in groups of up to five primary metabolites.

**Figure 1:**
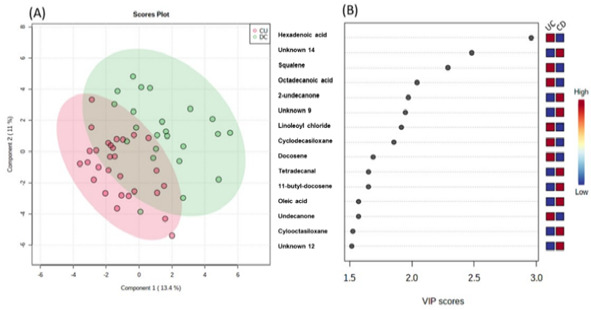
Patterns of separation between Crohn’s disease (CD) and ulcerative colitis (UC) by partial least squares discriminant analysis (PLS-DA). The green area corresponds to patients with CD, and the pink area corresponds to patients with UC. Variables in projection (VIP) score (B).

**Figure 1:**
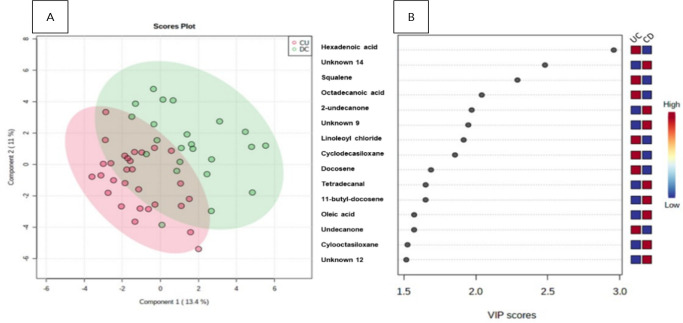
(A) Patterns of separation between Crohn’s disease and ulcerative colitis by partial least squares discriminant analysis. The green area corresponds to patients with Crohn’s disease, and the pink area corresponds to patients with ulcerative colitis. Variables in projection score (B).

UC: ulcerative colitis; CD: Crohn’s disease; VIP: variables in projection.

The VIP score classification of the components of the CD and UC groups is shown in Figure 1. It includes the most significant variations of metabolites that discriminate the UC and CD groups by the PLS-DA. Considering necessary VIP measurements above 2.0 as pre-established in the methodology of the work, the metabolites found were (highest score in the group UC): hexadecanoic acid, squalene, and octadecanoic acid. These three mentioned metabolites were used to discriminate the groups through the receiver operating characteristic (ROC) curve. The area under the curve (ACU) of the model was 0.765 (95% confidence interval [CI]: 0.35-1.0).

**Figure 2:**
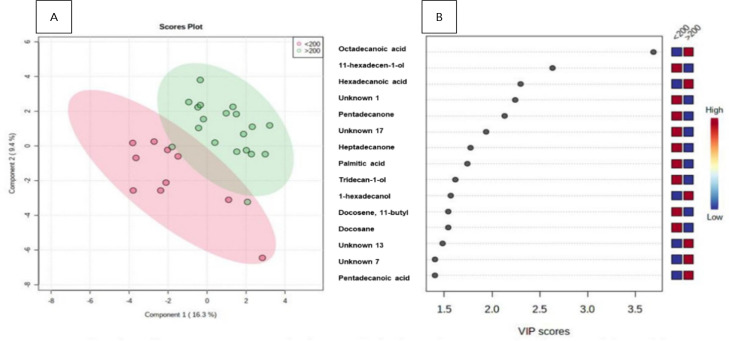
(A) Ulcerative colitis: patterns of separation between fecal calprotectin (<200 μc/g of feces vs. =200 μc/g of feces) by partial least squares discriminant analysis. The green area corresponds to patients with Crohn’s disease, and the pink area corresponds to patients with ulcerative colitis. Variables in projection score (B).

The relationship between the FC and the UC groups can be observed in Figure 2. The separation standards for the datasets showed good accuracy (0.65). The main metabolites found in this analysis were as follows: octadecanoic acid and hexadecanoic acid had the highest score in the group >200 μg/g, whereas 11-hexadecen-1 and pentadecanone had the lowest score in the group <200 μg/g. The ROC curve using such metabolites has resulted in an ACU of 0.77 within the confidence interval (95%CI 0.37-0.98).

VIP: variables in projection.

**Figure 3:**
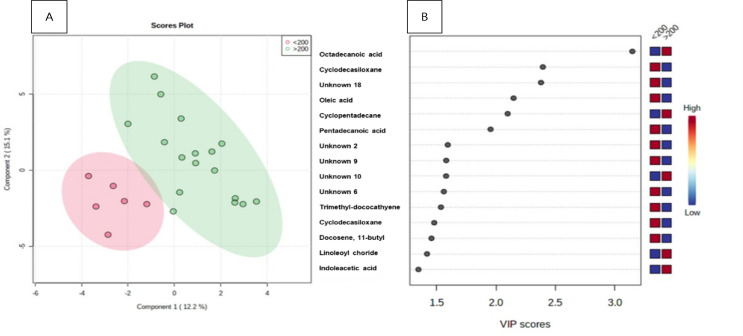
(A) Crohn’s disease: patterns of separation between fecal calprotectin (<200 μc/g of feces vs. =200 μc/g of feces) by partial least squares discriminant analysis. The green area corresponds to patients with Crohn’s disease, and the pink area corresponds to patients with ulcerative colitis. Variables in projection score (B).

Figure 3 shows metabolites that differentiate disease activity in CD and the separation standards for the datasets showed good accuracy (0.60). The main metabolites found in this analysis were: octadecanoic acid and cyclopentadecanoic acid demonstrated the highest score in the group >200 μg/g, whereas cyclodecasiloxone and oleic acid-1 had the highest score in the group <200 μg/g. The ROC curve, using such metabolites, shows an ACU of 0.85 within the confidence interval (95%CI 0-1). Octadecanoic acid, as in the CD group, is the most relevant metabolite in patients with disease activity.

VIP: variables in projection.

## DISCUSSION

This study has provided significant insights into the distinction of intestinal metabolites found in the feces of patients with CD or UC. Additionally, it corroborated that this composition is directly influenced by the degree of disease activity, as assessed through FC levels, a noninvasive biomarker widely employed in clinical practice.

Metabonomics serves as a quantitative approach for analyzing metabolites and establishing associations between metabolites and physiological as well as pathological changes^40^, rendering it a potent tool in the exploration of human health^9^. Metabolite analyses are commonly conducted utilizing noninvasive sample sources, such as fecal samples^24^, urine^41^, blood^11,20^, and tissue extracts^4^ not requiring invasive procedures.

In this investigation, fecal samples were subjected to analysis using mass spectrometry coupled with the GC-MS technique, leading to the identification of a distinct IBD metabolome signature. Discernible variations in metabolite patterns were observed between CD and UC patients. Specifically, the latter exhibited elevated levels of saturated long-chain fatty acids (SLCFAs) such as hexadecanoic acid, octadecanoic acid, triterpenoids, and squalene.

The differentiation of fecal metabolomics between individuals with IBD and healthy controls^17,18,23,25,35,37^ appears to be more conspicuous than the distinction between CD and UC subtypes. Studies conducted by Marchesi et al.^25^, Garner et al.^13^, and Bjerrum et al.^6^ successfully identified differences between CD and UC, akin to the findings in our study^6,13,25^. However, Santoru et al.^35^ and Franzosa et al.^12^ were unable to discern, via fecal metabolites, distinct characteristics between these two subtypes^12,35^.

The recognition of specific metabolites intricately linked with distinct disease states not only underscores a promising avenue for early detection and patient stratification but also heralds the potential to explore the mechanistic underpinnings of IBD. De Preter et al.^10^, while assessing the variation in VOCs in patients with active CD and UC (according to the Harvey-Bradshaw Index and Ulcerative Colitis Disease Activity Index, respectively), noted that 1-propanol was significantly higher in active CD, whereas styrene levels significantly and positively correlated with the UC Disease Activity Index (UCDAI). In our study, we also identified differences in the activity pattern based on the type of IBD: elevated levels of octadecanoic acid were seen in individuals with active CD, whereas in UC, higher levels of both octadecanoic acid and hexadecanoic acid were detected, with disease activity identified by increased FC levels.

Elevated levels of hexadecanoic acid or palmitic acid have been associated with inflammatory activity in various scenarios, such as metabolic dysfunction-associated fatty liver disease (MAFLD)^42^, astrocytes^29^, and particularly in intestinal cells, significantly impacting intestinal barrier integrity^15^. This alteration impairs paracellular permeability and cell-cell junctions and modifies cytokine expression profiles in intestinal epithelial cells^14^. Jansson et al.^18^ also observed higher levels of this metabolite, along with oleic acid, stearic acid, 6Z-, 9Z-, and 12Z-octadecatrienoic acid, linoleic acid, and arachidonic acid, in patients with ileal CD compared with colonic CD and healthy individuals, although these authors exclusively assessed CD patients. Our findings suggest that the elevation of palmitic acid may be directly related to the extent of lesion in UC patients, as active UC patients exhibited higher levels of this metabolite compared with those with lower inflammatory activity.

In contrast to the findings in this study, where octadecanoic acid or stearic acid emerged as a differentiating biomarker between CD and UC, and for increased activity in the latter, Tefas et al.^39^ identified that not only hexadecanoic acid but also its three isomers (stearyl palmitoleate, palmitoleyl stearate, and oleyl palmitate) were higher in the serum of patients with colonic CD than in those with UC.

Elevated fecal levels of stearic acid have also been observed in individuals with familial Mediterranean fever - an autoinflammatory disorder characterized by periodic attacks of acute inflammation and fever - as well as peptic ulceration^22^. This metabolite, along with heptanoic acid, constituted the most abundant evenand odd-numbered carbon SLCFAs in the colon lumen of a rat model of neonatal maternal separation. These SLCFAs promoted colonic muscle contraction and increased stool frequency in rats^43^. Collectively, these findings suggest that octadecanoic acid not only contributes to greater inflammatory activity but also plays a role in increased intestinal peristalsis, consequently stimulating diarrhea in UC patients.

Similarly to hexadecanoic acid and octadecanoic acid, squalene was a metabolite found in higher concentrations in individuals with UC compared to those with CD. Squalene, an insoluble isoprenoid compound, represents one of the intermediates in the cholesterol biosynthesis pathway, serving as a precursor for hormones, bile acids, and vitamin D^31^. It also exhibits antioxidant, anti-inflammatory, and drug carrier effects^21^. However, some studies have highlighted negative associations with this metabolite^27^. Elevated plasma levels of squalene have been observed in women with cardiovascular diseases^33^, correlating with visceral obesity^19^, implying that squalene could serve as a biomarker for cardiovascular diseases^30^.

Regarding intestinal cells, an intriguing study by Jiang et al.^19^ conducted on colon adenocarcinoma samples revealed that squalene synthase (an enzyme involved in squalene synthesis) accelerated colon cancer cell proliferation and promoted tumor growth. The inhibition of this enzyme, when combined with squalene epoxidase inhibition, resulted in a more pronounced suppressive effect on cell proliferation and tumor growth compared with single inhibition. In this context, the elevation of squalene, notably found in UC patients who are at a higher risk of developing colorectal cancer, might prove a valuable biomarker for the clinical monitoring of these individuals.

This study does present some limitations, such as the convenience sampling method and the absence of microbiota composition analysis. Additionally, certain unidentified VOCs found in the study remain beyond the scope of discussion and associations with IBD clinical implications. However, the significance of investigating fecal metabolites becomes evident due to their potential for distinguishing CD from UC, monitoring disease activity, and evaluating clinical progression in these patients. Furthermore, the identification of these metabolites opens a broad spectrum of possibilities for pharmacological and nonpharmacological therapeutic targets. However, given the intricate relationship between microbiota and fecal metabolites, intertwined with living conditions, dietary habits, and past and present history, it is imperative to explore diverse populations, thus forming a more robust database to inform effective clinical practices.

## CONCLUSIONS

In this context, distinctions in fecal metabolites were unearthed among patients with CD and UC, as well as those with heightened disease activity, as assessed by elevated FC levels, underscoring the potential of these metabolites in discriminating IBD subtypes and gauging disease severity. Collectively, these findings hold promise in informing therapeutic alternatives for IBD, including fecal microbiota transplantation and antioxidant therapy. However, several questions remain unanswered, given the presence of numerous unidentified compounds that might play significant roles in disease understanding. Consequently, further extensive investigations are imperative to shed light on these aspects in the future.

## Figures and Tables

**Figure:**
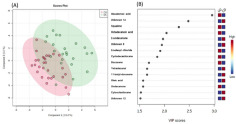
Patterns of separation between Crohn’s disease (CD) and ulcerative colitis (UC) by partial least squares discriminant analysis (PLS-DA). The green area corresponds to patients with CD, and the pink area corresponds to patients with UC. Variables in projection (VIP) score (B).

## Perspectives

This study shows that distinctions in fecal metabolites were unearthed among patients with CD and UC, as well as those with heightened disease activity, as assessed by elevated FC levels, underscoring the potential of these metabolites in discriminating IBD subtypes and gauging disease severity. Collectively, these findings hold promise in informing therapeutic alternatives for IBD, including fecal microbiota transplantation and antioxidant therapy.

## Central Message

The etiology of IBD is unknown, but the interaction between genetics, immunological, and environmental factors, such as microbiota, contribute to its development. In this context, intestinal dysbiosis - the imbalance of the intestinal microbiota - is closely related to the pathogenesis and clinical complications of IBD; however, it is still not possible to distinguish whether this dysbiosis is a cause or a consequence of the disease. In recent years, studies have aimed to identify laboratory markers of IBD, with high sensitivity and specificity for noninvasive diagnosis and monitoring of these diseases.
